# A Description of the Hemolytic Component in Sickle Leg Ulcer: The Role of Circulating miR-199a-5p, miR-144, and miR-126

**DOI:** 10.3390/biom12020317

**Published:** 2022-02-17

**Authors:** Edvan do Carmo Santos, Gabriela Imbassahy Valentim Melo, Paulo Vinícius Bispo Santana, Idaiara Graziele Silva Quadros, Sètondji Cocou Modeste Alexandre Yahouédéhou, Caroline Conceição da Guarda, Rayra Pereira Santiago, Luciana Magalhães Fiuza, Suéllen Pinheiro Carvalho, Elisângela Vitória Adorno, Carla Martins Kaneto, Teresa Cristina Cardoso Fonseca, Marilda Souza Goncalves, Milena Magalhães Aleluia

**Affiliations:** 1 Laboratório de Patologia Aplicada e Genética, Departamento de Ciências Biológicas, Universidade Estadual de Santa Cruz, Ilhéus 45662-900, BA, Brazil; edvanrih@gmail.com (E.d.C.S.); gabriela_melo00@hotmail.com (G.I.V.M.); vinibsantana13@gmail.com (P.V.B.S.); cmkaneto@uesc.br (C.M.K.); 2 Centro de Referência a Doença Falciforme de Itabuna, Itabuna 45600-075, BA, Brazil; idaiaragraziele@yahoo.com.br; 3 Laboratório de Investigação em Genética e Hematologia Translacional, Instituto Gonçalo Moniz, Fundação Oswaldo Cruz, Salvador 40296-710, BA, Brazil; modya006@yahoo.fr (S.C.M.A.Y.); cguarda4@hotmail.com (C.C.d.G.); rayrasantiago@hotmail.com (R.P.S.); mfiuza.luciana@gmail.com (L.M.F.); suellen.ufba@gmail.com (S.P.C.); mari@bahia.fiocruz.br (M.S.G.); 4 Laboratório de Pesquisa em Anemias, Departamento de Análises Clínicas e Toxicológicas, Faculdade de Farmácia, Universidade Federal da Bahia, Salvador 40170-115, BA, Brazil; liuadorno@hotmail.com; 5 Departamento de Ciências da Saúde, Universidade Estadual de Santa Cruz, Ilhéus 45662-900, BA, Brazil; teresacrfonseca@gmail.com

**Keywords:** sickle cell disease, sickle leg ulcer, microRNA, hemolysis, biomarkers

## Abstract

Sickle leg ulcers (SLU) are malleoli lesions with exuberant hemolytic pathophysiology. The microRNAs are potential genetic biomarkers for several pathologies. Thereby, we aimed to assess the expression of circulating miR-199a-5p, miR-144, and miR-126 in association with hemolytic biomarkers in SLU. This cross-sectional study included 69 patients with sickle cell disease, 52 patients without SLU (SLU-) and 17 patients with active SLU or previous history (SLU+). The results demonstrated elevated expression of circulating miR-199a-5p and miR-144 in SLU+ patients while miR-126 expression was reduced. Circulating miR-199a-5p and miR-144 were associated with hemolytic biomarkers such as LDH, indirect bilirubin, AST, GGT, iron, ferritin, RBC, hemoglobin, and NOm, in addition to association with impaired clinical profile of SLU. Furthermore, in silico analyses indicated interactions of miR-199a-5p with *HIF1A, Ets-1,* and *TGFB2* genes, which are associated with vasculopathy and reduced NO. In contrast, miR-126 was associated with an attenuating clinical profile of SLU, in addition to not characterizing hemolysis. In summary, this study demonstrates, for the first time, that hemolytic mechanism in SLU can be characterized by circulating miR-199a-5p and miR-144. The circulating miR-126 may play a protective role in SLU. Thus, these microRNAs can support to establish prognosis and therapeutic strategy in SLU.

## 1. Introduction

Sickle cell disease (SCD) is a common genetic disorder in Brazil, with 3500 new cases annually, and characterized by intravascular hemolysis, sterile inflammation, reduced nitric oxide (NO) bioavailability, and endothelial dysfunction [1]. The chronic hemolysis leads to vaso-occlusive episodes and clinical manifestations, such as painful crises, acute chest syndrome, stroke, and sickle leg ulcers (SLU) [2].

The SLU are cutaneous lesions with raised edges that frequently affect the malleolus of SCD patients [3]. SLU wound bed may present unviable tissues, such as necrotic tissue due to accumulation of dead cells or sloughy tissue without vascularization and constituted by cells fragments. In addition, the SLU wound bed may present viable granulation tissue with the proliferation of blood capillaries and epithelized tissue consisting of regenerated and dry epidermis [3,4].

The SLU occurrence has been associated with morbidity, work disability, social restriction, frequent recurrence, and recalcitration [2,5]. Thus, the evaluation of these factors is insufficient to understand the SLU clinical profile. Molecular biology techniques revealed the existence of microRNAs (miRNAs) which may modulate pathophysiology mechanisms [6]. miRNAs are small non-coding RNA molecules which act as positive or negative regulators in several pathologies, including SCD [6,7]. In this sense, miR-199a-5p has been associated with NO metabolism in vitro [8] and miR-144 may regulate fetal hemoglobin (HbF) production through gene silencing [6]. miR-126 has been associated with protective mechanisms against vascular damage in vitro and in vivo [9]. However, the role of these miRNAs in the intravascular hemolysis related to SLU occurrence is unknown.

Hence, this study aimed to investigate the expression of circulating miR-199a-5p, miR-144, and miR-126 in association with SLU occurrence as well as laboratory parameters of hemolysis.

## 2. Materials and Methods

### 2.1. Study Design

A cross-section study was performed in SCD patients, who were diagnosed at Itabuna Sickle Cell Disease Reference Center (CERDOFI), in the state of Bahia, Brazil. For inclusion criteria, patients with sickle cell anemia (SCA) or SC hemoglobinopathy (HbSC) confirmed, in steady state with absence of painful vaso-occlusive crises, hospitalization, infections or acute events, and which not received a blood transfusion for 3–4 months were included. All the other hemoglobin genotypes were excluded of the study.

Data regarding clinical manifestations were collected using a standardized and confidential questionnaire at the time of enrollment in the study and confirmed by the medical records at CERDOFI. This study was approved from the Santa Cruz State University (UESC) (protocol number: 11765319.7. 0000.5526) and was conducted in compliance with the ethical principles established by the revised Declaration of Helsinki. Furthermore, informed consent term was obtained from all subjects involved in the study.

### 2.2. Clinical Characteristics of SLU

The SLU were defined as cutaneous lesions in malleoli of SCD patients, which arose from a malleolar trauma, such as scratches and accidents, or spontaneously. The clinical description of active SLU, such as characteristics of wound bed and edges of lesions, recurrence episodes, and simultaneous SLU occurrence were performed by the data collected in medical record at CERDOFI.

### 2.3. Laboratory Biomarkers

Biochemical and hematological analyses were carried out at the Genetics and Applied Pathology Laboratory (LAPAGEN), UESC, as well as at Clinical Analyses Laboratory (LACTFAR), College of Pharmaceutical Sciences, Federal University of Bahia (UFBA).

The samples were collected from peripheral blood (15 mL) in closed system, as recommended by the Clinical and Laboratory Standards Institute (CLSI). The blood samples were distributed in tubes without anticoagulant with clot activator to obtain serum samples, and in tubes with anticoagulant ethylenediamine tetraacetic acid (EDTA) tripotassic acid (K3) to obtain plasma samples and total blood. Hematological analyses were performed using an automatic haematology analyzer ABX Pentra 80 (HORIBA Medical, Montpellier, France). Hemoglobin profiling was assessed by high-performance liquid chromatography using an HPLC/Variant II hemoglobin testing system (Bio-Rad, Hercules, CA, USA).

Serum levels of lactate dehydrogenase (LDH), total bilirubin and fractions, aspartate aminotransferase (AST), gamma-glutamyl transferase (GGT), and iron were assessed using an automatized equipment CB 400i (Wiener Lab, Rosario, Argentina). Ferritin levels were determined using an Architect i1000SR (Abbott, Chicago, IL, USA).

Nitric oxide metabolites (NOm) were determined in serum samples using Griess reagent, according to the method previously described [10], conducted at the Laboratory of Investigation in Genetics and Translational Hematology (LIGHT), Gonçalo Moniz Institute (IGM), Oswaldo Cruz Foundation (FIOCRUZ), Bahia, Brazil.

### 2.4. miRNA Analyses

Circulating microRNA expression analyses were performed at LAPAGEN, UESC. RNA molecules were isolated from serum and plasma samples and extraction was performed with TrizolLS (Invitrogen), according to manufacturer’s instructions. Total RNA was reverse transcribed using specific miR-primers and Taqman miRNA Reverse Transcription kit (Applied Biosystem, Waltham, MA, USA) according to the manufacturer’s instructions.

Circulating microRNAs hsa-miR-199a-5p, hsa-miR-126, and hsa-miR-144 were detected by RT-qPCR using Taqman MicroRNA assays (Applied Biosystems, Waltham, MA, USA) and a QuantStudio3 Instrument (ThermoFisher Scientific, Waltham, MA, USA) using standard thermal cycling conditions in accordance with manufacturer recommendations. PCR reactions were performed in a duplicate and experiments with coefficients of variation greater than 5% or that displayed unusual amplification curves were excluded from further analysis. A no-template control (NTC) and no reverse transcription controls (no-RT) were also included, as previously described [11,12].

The mean cycle threshold (Ct) values from duplicate measurements were used to calculate expression of target gene using 2^−ΔCt^ formula and present as fold change. miR-16, miR-U6, and miR-320 were analyzed to identify optimal endogenous reference genes in our set of samples. We validated that miR-320 could be used as a suitable reference in samples of our patients for RT-qPCR, based on the combination of four statistical approaches, as described in [11,12].

### 2.5. Statistical Analyses

Statistical analyses were conducted using the software program Statistical Package for the Social Sciences (SPSS) version 22.0 (IBM Software, New York, NY, USA). GraphPad Prism version 6.0 (Graphpad Software, San Diego, CA, USA) was used for graph assembly. Shapiro–Wilk test was used to determine quantitative variables distribution. The Mann–Whitney *U* test and independent t-test were used to compare the groups according to the normality of the distribution for each variable. Multivariate linear regression (MLR) analysis was performed to evaluate possible associations between hydroxyurea (HU) and miRNAs with regard to the outcome of interest, SLU. Pearson and Spearman correlation analyses were carried out in the preparation of heatmap graphs, demonstrating the strength of linear relationships between two quantitative variables with normal or non-normal distribution. Values of *p* < 0.05 were considered statistically significant.

Target genes were predicted in silico using miRWalk software (http://mirwalk.umm.uni-heidelberg.de/, accessed on 13 September 2021) with filter 90. To visualize and merge networks we used the Cytoscape software with miR targeting data and target gene interaction data. The Ontology Genes software (http://geneontology.org/, accessed on 13 September 2021) was used to run top ranked predicted genes and perform enrichment analysis on gene sets with a classification system to identify biological processes and reactome pathways associated with SCD.

## 3. Results

### 3.1. Characteristics of the Investigated Population

Sixty-nine SCD patients were included in this study, 42% (29/69) with SCA and 58% (40/69) with HbSC. Seventy-five percent (52/69) of the patients did not present SLU (SLU-), while 25% (17/69) were presenting active SLU or reported a previous history (SLU+). Among SLU+ patients, 76.5% (13/17) had active SLU, while 23.5% (4/17) had healed SLU at study enrolment. Furthermore, there were 30.7% (16/52) SLU- patients with HbSS genotype, and 69.3% (36/52) with HbSC genotype. For SLU+ patients, 76.5% (13/17) had HbSS genotype while 23.5% (4/17) had HbSC genotype.

In addition, 82.3% (14/17) SLU+ patients and 53.8% (28/52) SLU- patients were taking HU at least two times a day. HU is indicated for the treatment of sickle cell disease (SCD) and was approved in 1999 by the U.S. Food and Drug Administration.

### 3.2. Circulating miRNAs Expression in SCD Patients and between HbSS and HbSC Genotype with and without SLU

**Figure 1:**
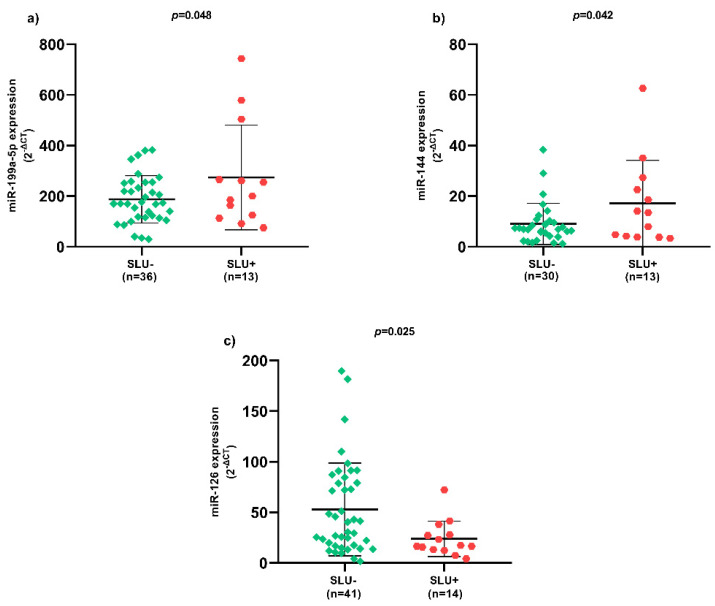
Expression of circulating miRNAs between SLU- and SLU+ patients. (**a**) Expression of circulating miR-199a-5p between SLU- and SLU+ patients; (**b**) Expression of circulating miR-144 between SLU- and SLU+ patients; (**c**) Expression of circulating miR-126 between SLU- and SLU+ patients. SLU-, patients without sickle leg ulcer; SLU+, patients with active sickle leg ulcer or previous history; miR, microRNA; *p*-value obtained using independent *t*-test.

Regarding SLU, there was higher miR-199a-5p (Figure 1a) and miR-144 (Figure 1b) expression, as well as lower miR-126 (Figure 1c) expression in SLU+ patients (*p* < 0.05).

In SLU- patients with HbSC genotype, there was higher expression of circulating miR-199a-5p, miR-144, and miR-126 (Figure S1). In addition, circulating miR-126 expression was higher in SLU+ patients with HbSC genotype (Figure S1).

### 3.3. Correlation Coefficients between Hemolytic Biomarkers and Circulating miRNAs Expression in SLU+ Patients

**Figure 2:**
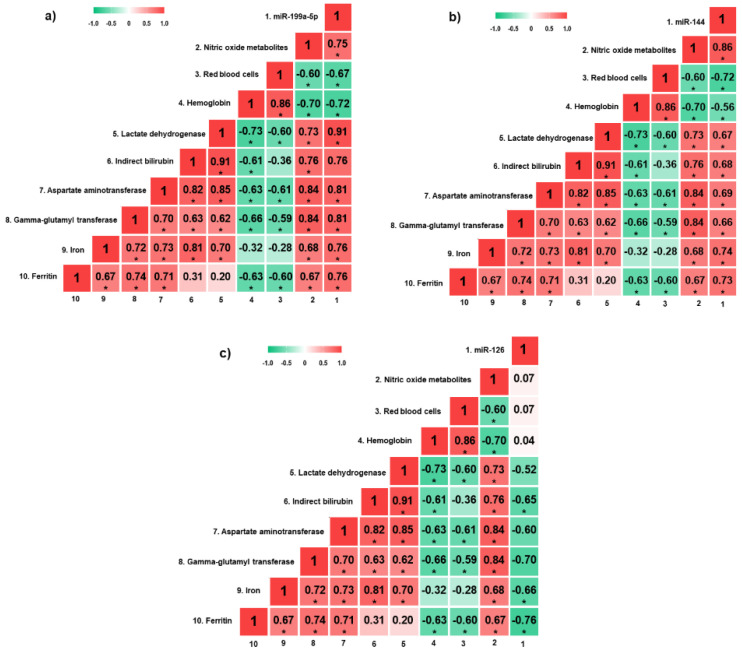
Heatmap of correlation coefficients between hemolytic biomarkers and circulating miRNAs in SLU+ patients. (**a**) Correlation analyses between hemolytic biomarkers and circulating miR-199a-5p; (**b**) correlation analyses between hemolytic biomarkers and circulating miR-144; (**c**) correlation analyses between hemolytic biomarkers and circulating miR-126. miRNAs, microRNAs; SLU+, patients with active sickle leg ulcer or previous history. * *p* < 0.05. *p*-value obtained by Pearson correlation.

miR-199a-5p expression was positively correlated with NOm, LDH, AST, GGT, iron, and ferritin levels, and negatively correlated with red blood cell (RBC) count and hemoglobin levels (*p* < 0.05) (Figure 2a). Moreover, there were positive correlations between miR-144 expression and NOm, LDH, indirect bilirubin, AST, GGT, iron, and ferritin levels, as well as negative correlations with RBC count and hemoglobin levels (*p* < 0.05) (Figure 2b). Regarding miR-126 expression, there were negative correlations with indirect bilirubin, iron and ferritin levels (*p* < 0.05) (Figure 2c).

### 3.4. Circulating miRNAs Expression in Active SLU

**Figure 3:**
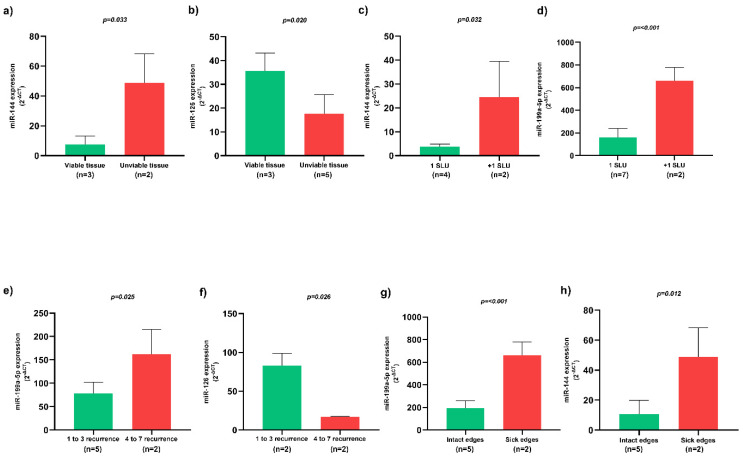
Association of circulating miRNAs and clinical characteristics of active SLU. (**a**) Expression of circulating miR-144 in SLU with viable and unviable tissues; (**b**) Expression of circulating miR-126 in SLU with viable and unviable tissues; (**c**) Expression of circulating miR-144 in patients with one and more than one SLU; (**d**) Expression of circulating miR-199a-5p in patients with one and more than one SLU; (**e**) Expression of circulating miR-199a-5p in patients with multiple SLU recurrence; (**f**) Expression of circulating miR-126 in patients with multiple SLU recurrence; (**g**) Expression of circulating miR-199a-5p in SLU with intact and sick edges; (**h**) Expression of circulating miR-144 in SLU with intact and sick edges. SLU, sickle leg ulcers; miR, miRNA. 1 SLU, one sickle leg ulcer; +1 SLU, more than one sickle leg ulcer; *p*-value obtained using independent *t*-test.

Patients whose SLU wound bed was constituted by unviable tissues, such as necrotic and sloughy tissues, presented higher miR-144 expression (Figure 3a) and lower miR-126 expression (Figure 3b) (*p* < 0.05). Patients with more than one SLU simultaneously presented higher miR-144 (Figure 3c) and miR-199a-5p (Figure 3d) expression (*p* < 0.05). In addition, SLU recurrence between 4 to 7 episodes presented higher miR-199a-5p expression (Figure 3e) and lower miR-126 expression (Figure 3f) (*p* < 0.05). SLU with sick edges were associated with higher miR-199a-5p (Figure 3g) and miR-144 (Figure 3h) expression (*p* < 0.05).

### 3.5. Association of HU with Circulating miRNAs Expression in Patients with and without SLU

Two MLR models were performed with HU as dependent variable. In SLU- patients, miR-199a-5p and miR-126 expression were independently associated with HU (R^2^ = 0.486; *p* < 0.05), while in SLU+ patients only miR-126 expression presented independent association (R^2^ = 0.779; *p* < 0.05) (Table 1).

### 3.6. Target Gene Prediction with Biological Processes of miR-199a-5p to SCD Patients

**Figure 4:**
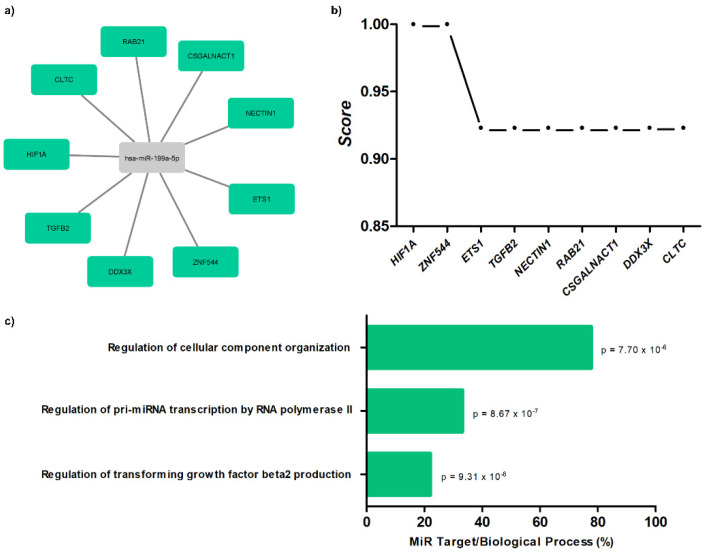
Target gene prediction with biological processes of miR-199a-5p. (**a**) Interaction networks of miR-199a-5p and target genes using miRWalk analysis; (**b**) correlation between level significance by score for genes; (**c**) biological processes for miR-199a-5p with *p*-values.

The results demonstrate interaction networks of miR-199a-5p and target genes using miRWalk analysis (Figure 4a). Correlation between level significance by the score for these genes is presented (Figure 4b), as well as biological processes (Figure 4c; Table S1).

## 4. Discussion

Previous analyses suggested that intravascular hemolysis has a direct association with the occurrence, recurrence, and clinical severity of the SLU lesions [2,13,14]. However, due to the genetic and clinical heterogeneity of SCD, the precise role of hemolytic process is still not fully understood, emphasizing the need to analyze new biomarkers that aid understanding, supporting better prognosis and therapeutic strategy.

This study demonstrates differential expression of circulating miR-199a-5p, miR-144, and miR-126 in association with the occurrence of SLU. In SLU+ patients there was a higher expression of miR-199a-5p and miR-144. These miRNAs were positively correlated to classical biomarkers in intravascular hemolysis, such as LDH, indirect bilirubin, AST and ferritin, in addition to negative correlations with RBC count and hemoglobin levels. Thus, these associations suggest that circulating miR-199a-5p and miR-144 may act as genetic biomarkers integrated into the hemolytic process evident in SLU, possibly positively regulating this pathophysiology.

Furthermore, positive correlations between the expression of circulating miR-199a-5p and miR-144 with serum levels of GGT and iron in SLU+ patients suggest the involvement of the biliary tract resulting from hemolysis with hyperbilirubinemia and vascular dysfunction, respectively [13,14]. These results extend the role of circulating miR-199a-5p and miR-144 as biomarkers in intravascular hemolysis in SLU.

Regarding the clinical profile of SLU, associations of high circulating miR-199a-5p and miR-144 with simultaneous occurrence of SLU, episodes of recurrence, and tissue severity with infiltration of SLU wound beds by non-viable tissues, and injured edges suggest that these miRNAs may be potential markers of the clinical evolution of these lesions.

Concerning changes in NO metabolism, previous analyses indicated that the expression of miR-199a-5p negatively regulates endothelial NO production in vitro [15], in addition to inhibiting the phosphorylation of the endothelial nitric oxide synthase (eNOS) responsible for NO reduction [8]. However, studies on the interactions of circulating miR-199a-5p and miR-144 in NO metabolism in SLU are not known. Thus, the present study showed positive correlations between the expression of circulating miR-199a-5p and miR-144 with serum levels of NOm, suggesting that there might be biological relevance for increased expression of miR-199a-5p and miR-144 and endothelial dysfunction and vasculopathy related to the occurrence of SLU.

In contrast, lower expression of circulating miR-126 in the present study suggests a different context. Previous observations have indicated that this miRNA may play a protective role against hypoxia/reoxygenation-induced vascular damage [9,16]. miR-126 has also been associated with erythropoiesis inhibition and maintenance of vascular integrity [16], despite its role in SLU remains unknown. Our analyses demonstrated reduced expression of circulating miR-126 in SLU+ patients and elevated expression in HbSC patients, which is known for lower frequency of SLU [2], in addition to negative associations with hemolytic biomarkers, such as indirect bilirubin, iron and ferritin, and clinical evolution of SLU, such as infiltration of viable tissues in the wound beds, for example, highly vascularized granulation tissue and low frequency of SLU recurrence. Therefore, we hypothesize that the expression of circulating miR-126 may exert protective mechanisms in SLU, although the specific role is not fully understood.

Furthermore, target gene analyses for circulating miR-199a-5p suggest relevant interactions in the SCD pathophysiology about *HIF1A, Ets-1,* and *TGFB2* genes. In SCD, *HIF1A* has been associated with transcription of regulatory pathways in tissue hypoxia and ischemia [17]. The *Ets-1* gene has been observed to have transcriptional gene action in the in vitro production of NO [18]. The *TGFB2* has been described as a target of miRNAs in promoting angiogenesis, healing, and vascular protection [19,20]. Besides, miR-199a-5p can inhibit the expression of *HIF1A* and *Ets-1* in malignant pathologies [21,22]. Therefore, our in silico analyses allows us to suggest possible regulatory mechanisms of circulating miR-199a-5p for the occurrence of SLU, all previously associated with the hemolytic process, such as tissue ischemia and reduced NO bioavailability [4,23].

With respect to treatment in SCD, HU is the main pharmacological therapy for SCD, known for increase HbF concentrations and reduce the hemolytic anemia [14]. However, the association between SLU occurrence and HU is still misunderstood [2,14]. In this study, the MLR model demonstrated the associating of the circulating miRNAs and HU in SLU+ patients. The specific association of miR-126 and HU in SLU+ patients is controversy due to protective role of HU and miR-126 in vascular damage [9,16,18]. These results show the need of new studies for support the precise HU role in SLU occurrence.

In summary, this study demonstrates, for the first time, that circulating miRNAs miR-199a-5p and miR-144 may be part of the genetic background responsible for hemolytic pathophysiology in the occurrence and clinical evolution of SLU. The association of circulating miR-126 expression with attenuating clinical profile suggests a protective role for SLU occurrence. Importantly, these preliminary results indicate that these miRNAs can act as potential biomarkers of prognosis and therapeutic strategy in patients with SLU, in addition to providing support for additional studies involving analysis of miRNAs in the hemolytic process associated with the occurrence of SLU.

## Figures and Tables

**Table 1:** Multivariate linear regression models of hydroxyurea in association with confounding variables.

	Independent Variables	Dependent Variable	*p*-Value	β	R^2^	*p*-Value of the Model
**SLU- patients** **N = 43**	Circulating miR-199a-5p	**Hydroxyurea**	**0.007**	0.695	**0.486**	**0.022**
	Circulating miR-144		0.096	0.468		
	Circulating miR-126		**0.009**	−0.834		
**SLU+ patients** **N = 15**	Circulating miR-199a-5p		0.485	0.191	**0.779**	**0.043**
	Circulating miR-144		0.997	−0.001		
	Circulating miR-126		**0.011**	−0.885		

SLU-, patients without sickle leg ulcers; SLU+, patients with active sickle leg ulcers or previous history; miR, microRNA; R^2^: coefficient of determination; β: coefficient of regression; Bold *p*-values indicate significance at *p* < 0.05.

## Data Availability

Data are available on request to the corresponding author.

## Supplementary Materials

The following supporting information can be downloaded at: https://www.mdpi.com/article/10.3390/biom12020317/s1, Figure S1: Expression of circulating miRNAs in SLU- and SLU+ patients with HbSS and HbSC genotype. Table S1: Biological processes for miR-199a-5p.
