# Supplementary material for: A Description of the Hemolytic Component in Sickle Leg Ulcer: The Role of Circulating miR-199a-5p, miR-144, and miR-126
Source: Biomolecules. 2022 Feb 17;12(2):317. doi: 10.3390/biom12020317 (PMC8869177; doi:10.3390/biom12020317)
Supplement: Supplementary file 1 [file biomolecules-12-00317-s001.zip › biomolecules-1564996-supplementary.pdf]

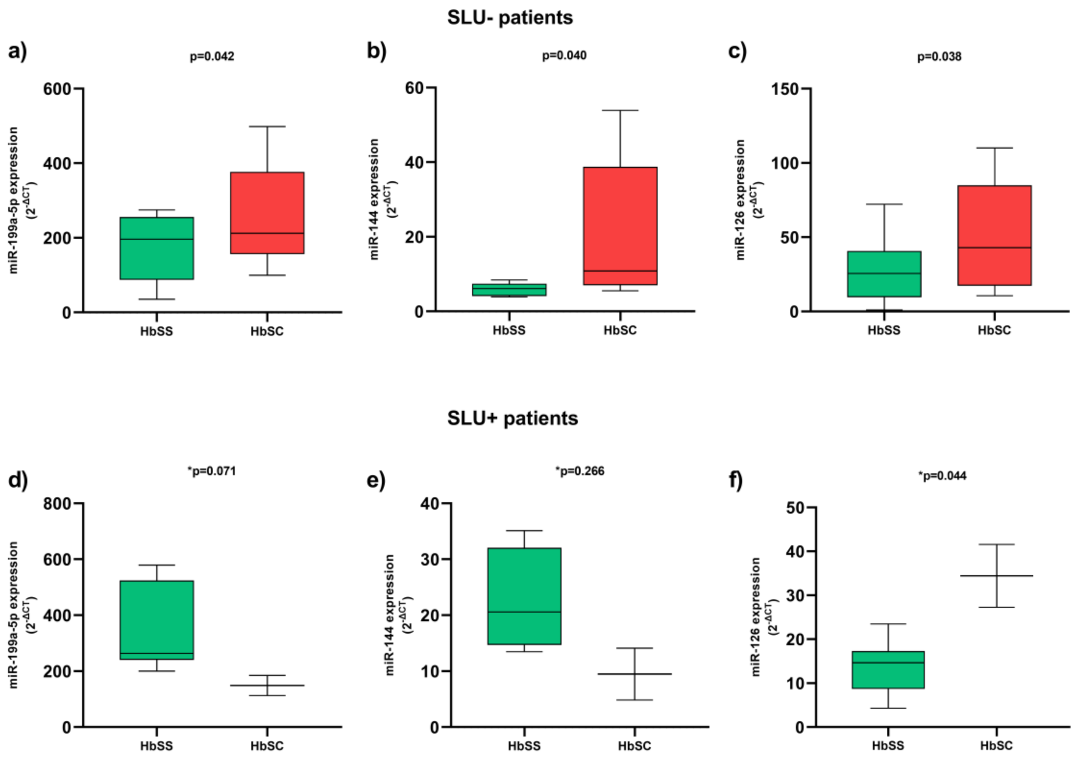

Figure S1. Expression of circulating miRNAs in SLU- and SLU+ patients with HbSS and HbSC genotype.

Table S1. Biological processes for miR-199a-5p.

Biological processes	Accession	Genes
Regulation of transforming growth factor beta2 production	GO:0032909	*TGFB2, HIF1A*
Regulation of pri-miRNA transcription by RNA polymerase II	GO:1902893	*TGFB2, HIF1A, ETS1*
Regulation of cellular component organization	GO:0051128	*TGFB2, HIF1A, ETS1, RAB21, DDX3X, NECTIN1, CLTC*
